# Right Ventricular Perforation Leading to Sudden Death Due to Extracorporeal Membrane Oxygenation (ECMO) Canula Dislodgement

**DOI:** 10.7759/cureus.39177

**Published:** 2023-05-18

**Authors:** Freny Sebastian, Arshan A Khan, Dilshan Dhillon, Amir Kaki

**Affiliations:** 1 Internal Medicine, Ascension St. John Hospital, Detroit, USA; 2 Cardiology, Ascension St. John Hospital, Detroit, USA

**Keywords:** leading cardiac arrest, acute respiratory distress syndrome, ventricular perforation, complication, extracorporeal membrane oxygenation, covid-19

## Abstract

ECMO has been playing an increasingly important role in the management of coronavirus disease (COVID-19)-related acute respiratory distress syndrome (ARDS). However, despite its potential benefits, high mortality rates are still being reported worldwide. Herein, we report the case of a 32-year-old male who presented with worsening shortness of breath secondary to COVID-19. Unfortunately, he experienced a sentinel event when the cannula became dislodged due to coughing, which led to a right ventricular perforation and sudden pulseless electrical activity (PEA) cardiac arrest.

## Introduction

Venovenous extracorporeal membrane oxygenation (VV ECMO) is a life-saving therapy used in critically ill patients with respiratory failure. It involves the use of a membrane oxygenator and a pump to remove deoxygenated blood from the patient, oxygenate it, and return it to the patient's body. This technique can provide temporary support for patients with severe respiratory failure who have not responded to conventional therapies. VV ECMO has been shown to improve survival rates in patients with acute respiratory distress syndrome (ARDS), influenza-associated respiratory failure, and coronavirus disease 2019 (COVID-19)-associated respiratory failure [1-2]. However, VV ECMO is a complex therapy that requires specialized equipment and trained staff. Therefore, careful patient selection, optimal timing, and management are crucial to ensuring the best possible outcomes [1].

Although VV ECMO can be lifesaving, it is also associated with several potential complications. Bleeding, infection, thrombosis, and hemolysis are some of the most common complications. Other less common complications include air embolism, pump failure, and circuit rupture. The incidence and severity of these complications can vary depending on patient characteristics, the underlying disease, and the management of VV ECMO [2].

In this case report, we are presenting a rare case of sudden death due to right ventricular perforation resulting from VV ECMO cannula dislodgement.

## Case presentation

The patient, in this case, was a 32-year-old male with no past medical history who had presented to an outside medical facility with shortness of breath. He was diagnosed with COVID-19 pneumonia, which led to acute hypoxic respiratory failure and required intubation. The patient was then transferred to our facility for VV ECMO evaluation. 

Upon arrival, the patient's initial vitals were significant for sinus tachycardia with a heart rate of 109 beats per minute and hypoxia with an oxygen saturation of 70% on 100% fraction of inspired oxygen (Fio2), and positive end-expiratory pressure (PEEP) of 12. All other vital signs were within normal limits. Physical examination revealed coarse bilateral breath sounds and trace pitting edema of the lower extremities. The laboratory results on admission showed leukocytosis with a white blood cell count (WBC count) of 12.5 K/MCL, thrombocytopenia with a platelet count of 42 k/mcl, an elevated D-Dimer level of more than 20,000 ngFEU/mL, and elevated liver enzymes with an aspartate aminotransferase of 48 IUnits/L and an alanine transaminase of 62 unit/L.

The patient was evaluated by our ECMO team due to worsening hypoxemia despite maximum ventilator support and was subsequently cannulated for VV ECMO via the right internal jugular approach with a 31 French dual lumen cannula devise to a Tandem Lung oxygenator and LifeSPARC pump. The patient was then extubated to help attenuate lung barotrauma and promote upright positioning and early ambulation. From a respiratory standpoint, the patient's condition began to improve. However, he developed frequent episodes of panic and coughing attacks, which were managed with dextromethorphan, fentanyl, and lorazepam as needed. The increased metabolic demand from coughing episodes caused low oxygen saturation levels, which improved with low-dose fentanyl.

**Figure 1:**
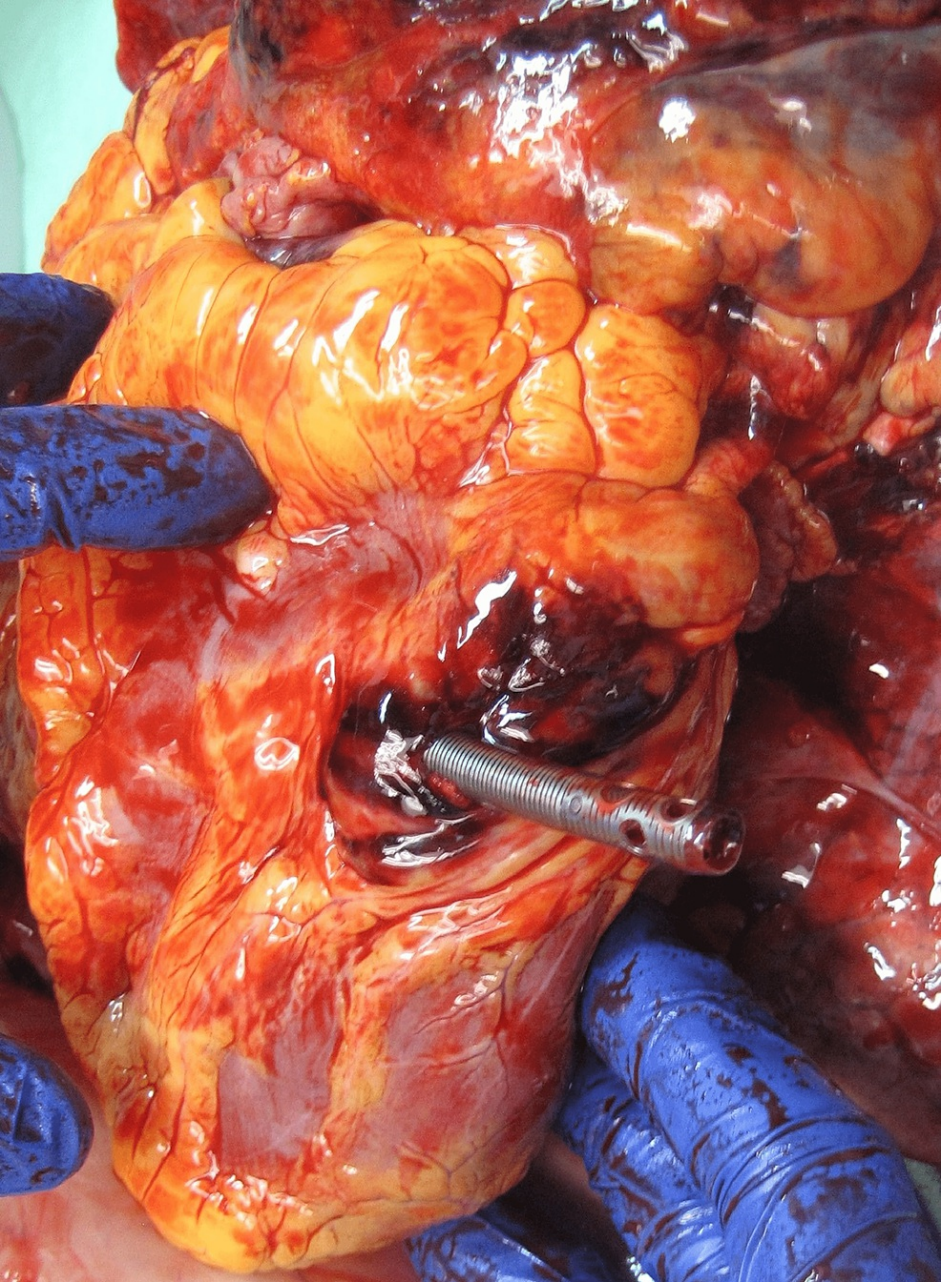
Autopsy findings show right ventricular perforation from venovenous extracorporeal membrane oxygenation (VV ECMO) canula dislodgement

In the days before planning decannulation, the patient started coughing and suddenly developed cyanosis from his neck up, followed by pulseless electrical activity (PEA) cardiac arrest. Cardiopulmonary resuscitation (CPR) was initiated, and the pump was turned down. There were no issues with the oxygenator or pump. A post-mortem autopsy was done with consent from the patient's family, and it revealed right ventricular perforation (Figure 1). The cannula had to be re-secured earlier that night, as it had migrated back. It likely fell into the right ventricle when he was coughing with severe intrathoracic pressure changes, causing a perforated RV-free wall.

## Discussion

VV ECMO is a form of extracorporeal life support that provides mechanical support to the lungs in patients with severe respiratory failure. It is a complex and invasive intervention that is typically reserved for patients with refractory hypoxemia or hypercarbia despite maximal medical management [3].

VV ECMO is commonly used in patients with ARDS who fail conventional mechanical ventilation. ARDS is a life-threatening condition that leads to the development of severe hypoxemia, and VV ECMO can provide support while allowing the lungs to heal. In addition to ARDS, VV ECMO has been used in other respiratory conditions, such as severe pneumonia, acute exacerbation of chronic obstructive pulmonary disease (COPD), and acute respiratory failure in the setting of pulmonary embolism or acute lung injury [2].

Despite its potential benefits, VV ECMO is associated with significant risks and potential complications. The most common complications of VV ECMO include bleeding, infection, thrombosis, and mechanical complications such as circuit failure, air embolism, and oxygenator failure [4]. Bleeding is a particularly concerning complication, as anticoagulation is required to maintain circuit patency but increases the risk of bleeding. Neurological complications are also a concern, including stroke and seizures, and can occur due to embolization or decreased cerebral perfusion [4].

Several studies have evaluated the use of VV ECMO in patients with severe respiratory failure. One retrospective cohort study found that VV ECMO was associated with improved survival in patients with severe ARDS compared to conventional mechanical ventilation [4]. Another study found that early initiation of VV ECMO in patients with severe ARDS was associated with improved survival and a reduced duration of mechanical ventilation [5]. However, a randomized controlled trial (the EOLIA trial) found that VV ECMO did not significantly improve survival in patients with severe ARDS compared to conventional mechanical ventilation [6].

Right ventricular perforation is a rare but serious complication that can occur during VV ECMO. This complication can lead to hemodynamic instability, cardiac tamponade, and even death. The incidence of right ventricular perforation during VV ECMO ranges from 0.2% to 1.9% [4]. The most common cause of right ventricular perforation is cannula malposition or migration, which can lead to direct injury to the right ventricle. Other potential causes of right ventricular perforation include high levels of anticoagulation, the use of large-bore cannulas, and underlying cardiac pathology.

Patients with right ventricular perforation may present with a sudden onset of chest pain, hemodynamic instability, and cardiac tamponade. Immediate intervention is required to address the perforation and prevent further complications. Treatment options include pericardiocentesis, surgical repair, or conversion to veno-arterial ECMO for additional cardiac support.

Several strategies can be employed to reduce the risk of right ventricular perforation during VV ECMO, including careful cannula placement and securement, frequent monitoring of cannula position, avoidance of high levels of anticoagulation, and consideration of alternative cannulation strategies such as bicaval dual-lumen cannulas.

Right ventricular perforation is a rare but potentially life-threatening complication that can occur during VV ECMO. Awareness of the risk factors and prompt recognition and intervention are essential for optimizing patient outcomes.

## Conclusions

In summary, VV ECMO is a potentially life-saving intervention for patients with severe respiratory failure. However, it is associated with significant risks and potential complications such as right ventricular perforation, cardiac tamponade, and bleeding. The decision to initiate VV ECMO should be made on a case-by-case basis, weighing the potential risks and benefits for each patient. Close monitoring and management of potential complications are crucial to ensuring the best possible outcomes for patients undergoing VV ECMO.
